# Nutritional Status of Iodine in a Group of Pregnant Women from the State of Minas Gerais Correlated with Neonatal Thyroid Function

**DOI:** 10.1055/s-0042-1756147

**Published:** 2022-11-29

**Authors:** Natália Campos Gonçalves Scherr, Anelise Impelizieri Nogueira, Kamilla Maria Araújo Brandão Rajão, Henrique Vitor Leite

**Affiliations:** 1 Departamento de Ginecologia e Obstetrícia, Universidade Federal de Minas Gerais, Belo Horizonte, MG, Brazil

**Keywords:** urinary iodine, iodine sufficiency, iodine, pregnancy, iodúria, suficiência iódica, iodo, gestação

## Abstract

**Objective** To evaluate the iodine sufficiency of pregnant women assisted in a University Hospital of Minas Gerais, and to correlate the urinary concentrations of maternal iodine with the fetal thyroid hormone levels at birth.

**Methods** Urinary iodine concentrations from 30 pregnant women with a singleton pregnancy and gestational age lower than 20 weeks were analyzed. Occasional samples of the mothers' urine were collected for the urinary iodine concentration dosage, and these were correlated with the newborns' thyroid-stimulating hormone (TSH) levels.

**Results** The median iodine urinary concentration of this study's pregnant women population was 216.73 mcg/l, which is proper for the group, following the World Health Organization (WHO). No cases of neonatal hypothyroidism were reported in the study, which corroborates the iodine sufficiency in this population sample.

**Conclusion** This study shows that despite the increased demand for iodine from pregnant women and the Brazilian Health Regulatory Agency (ANVISA) recommendation of 2013 for reduction of salt iodization levels, the population of pregnant women attended in the prenatal ambulatory of normal risk from the Federal University of Minas Gerais is considered sufficient in iodine. As a higher sample is necessary for the confirmation of these findings, it is too early to recommend the universal supplementation of iodine for Brazilian pregnant women, and more studies must be carried out, considering that iodine supplementation for pregnant women in an area of iodine sufficiency is associated to the risks of the fetus's excessive exposure to iodine.

## Resumo

**Objetivo** Avaliar a suficiência iódica de gestantes atendidas em um Hospital Universitário de Minas Gerais e correlacionar as concentrações urinárias de iodo materno com os níveis de hormônios tireoidianos fetais ao nascimento.

**Métodos** Foi analisada a concentração urinária de iodo de 30 gestantes com gestação única e idade gestacional menor que 20 semanas. Foram coletadas amostras ocasionais de urina materna para dosagem da concentração urinária de iodo, e estas foram correlacionadas com os níveis de hormônio estimulante da tiroide (TSH) dos recém-nascidos.

**Resultados** A mediana da concentração urinária de iodo das gestantes estudadas foi de 216,73 mcg/L, sendo adequada para o grupo segundo a Organização Mundial de Saúde (OMS). Não houve nenhum caso de hipotireoidismo neonatal no estudo, o que corrobora a suficiência de iodo nesta amostra populacional.

**Conclusão** Esse estudo demonstra que apesar do aumento da demanda de iodo pelas gestantes e da recomendação da Agência Nacional de Vigilância Sanitária (ANVISA) de 2013 da redução dos níveis de iodação do sal, a população de gestantes atendidas no ambulatório de pré-natal de risco habitual da Universidade Federal de Minas Gerais é considerada suficiente em iodo. Apesar de uma maior amostragem ser necessária para a confirmação destes achados, é cedo para recomendar a suplementação universal de iodo para as gestantes brasileiras e mais estudos precisam ser realizados, levando-se em conta que a suplementação de iodo para gestantes em áreas suficientes em iodo está associada aos riscos da exposição excessiva de iodo ao feto.

## Introduction

Thyroid dysfunctions during pregnancy result in higher maternal and fetal morbidity. One of the causes of thyroid dysfunction is the deficiency or excess of iodine. During pregnancy, there is an increase in the production of thyroid hormones, a significant increment of kidney clearance, and an increase of the (T4) thyroxine turnover through placental type 3 deiodinase in the second half of the pregnancy, thus occasioning an increased need for iodine. 1 2

Over the last years, international consensus on thyroid diseases during pregnancy have recommending the iodine supplementation for pregnant women. These recommendations appeared based on studies that showed iodine insufficiency in pregnant women originated from populations with proper iodine intake. 3 4 5 However, there is a lack of Brazilian studies on this subject.

Iodine deficiency has multiple effects on the growth and development of animals and humans. Studies in animal models show that iodine deficiency in mothers and, consequently, the lack of thyroid hormones causes goiter, cortical, and cerebellar changes in newborns. 6 However, the excess of iodine may also lead to an increase in the incidence of hypothyroidism and hyperthyroidism in susceptible subjects. 3 7

Despite iodine being an essential nutrient to produce thyroid hormones, the excess of this substance inhibits three processes in the production of thyroid hormones: iodine capture, iodization of thyroglobulin (Wolff-Chaikoff effect), and the release of thyroid hormones from the gland. This way, the excess of iodine causes a transitory inhibition of the thyroid hormones' synthesis. However, from 2 to 4 weeks of continued exposure to iodine excess, organification and the synthesis of thyroid hormone return to normal, probably due to a decrease in the NIS activity, the sodium-iodide symporter, a carrier protein of iodine that captures iodine to the interior of the thyroid cell. 8 Some subjects cannot escape from this effect, and the iodine excess triggers the hypothyroidism. 9 Fetuses are particularly susceptible, as this escape ability is not totally mature until 36 weeks of pregnancy. 4

The best parameter to evaluate the nutritional status of the population is the urinary iodine concentration. 10 The absorption of iodide in healthy adults is higher than 90%, and more than 90% of the ingested iodine is released in the urine. 11

The thyroid-stimulating hormone (TSH) in newborns is also a great indicator of iodine deficiency. The thyroid in newborns has a low input of iodine compared to adults, so the iodine turnover is much larger. This high turnover is exaggerated in iodine deficiency and requires a high stimulation from the TSH. 10 11 12 The increase in the number of newborns with moderate elevation of TSH concentrations (above 5 mIU/l) is proportional to the level of iodine deficiency during pregnancy. A frequency of < 3% of neonatal TSH values > 5 mIU/l indicates iodine sufficiency in a population. 13

We carried out this study to evaluate the nutritional status of iodine in a sample of pregnant women from the state of Minas Gerais, Brazil, correlating these values with the thyroid function of newborns.

## Methods

This was an observational study in a population of healthy pregnant women, all of whom attended for a first elective appointment in the prenatal ambulatory of normal risk from the Federal University of Minas Gerais in the period from May to December 2017. These pregnant women were invited to participate in the study, informed on its purpose, and received an informed consent term, in compliance with the recommendations of the Declaration of Helsinki. Pregnant women with a singleton pregnancy and gestational age of up to 20 weeks were included. Patients with a history of thyroid disease and/or using multivitamins containing iodine or food supplements were excluded. The patients answered questionnaires regarding age, gestational age, parity, use of multivitamins and food supplements, use of drugs, smoking habits, alcoholism, and family history of thyroid diseases. Pregnant women were also subjected to height and weight measurements. Initially, 39 patients were recruited; however, 9 of them did not present the inclusion criteria of the study. Therefore, 30 patients were enrolled. Generally, according to World Health Organization (WHO), thirty urinary iodine determinations in a defined sampling group are sufficient for the interpretation about iodine sufficiency. 10

During the recruitment, a single sample of urine was collected in the afternoon for urinary iodine dosage. The samples were stored in a freezer at −20° C until the dosage time. It is known that freezing does not interfere with sample stability. 14 The urinary iodine concentration measurement (in mcg/L) of occasional aliquots is considered as the real laboratory biochemical parameter for iodine use by the community. 15 Urinary iodine concentration was determined using inductively coupled plasma mass spectrometry. The manufacturer's reference interval was from 70 to 530 μg/g of creatinine. The iodine status in pregnant women at population level was classified according to the WHO, UNICEF, and ICCIDD guidelines from 2007; with the median urinary iodine measures being < 150 mcg/l considered as insufficient iodine intake; from 150 to 240 mcg/l as adequate iodine intake; from 250 to 249 mcg/k as more than adequate; and ≥ 500 mcg/l as excessive intake. 10

The results of neonatal TSH measurements were provided by the Núcleo de Ação e Pesquisa em Apoio Diagnóstico (NUPAD). The quantitative determinations of TSH were made in blood samples dried in filter paper, using the automatic system of fluoroimmunoassay 1235 AutoDELFIA (PerkinElmer Inc., Waltham, MA, USA). The cut-off values recommended by the manufacturer are: < 9 µIU/ml for normal values; from 9 to 18 µIU/ml for borderline; and  > 18 µIU/ml for hypothyroidism.

The statistical analyses were made using the R (R Foundation for Statistical Computing, Vienna, Austria) software, version 3.6.1.

In the descriptive analysis of categorical variables (qualitative variables), the absolute and relative frequencies were used. In the description of numerical variables, measurements of position, central trend, and dispersion were used.

Initially, to relate the urinary iodine concentration of pregnant women with the variables, a univariate analysis was made. The Mann Whitney test was used for the categorical variables with two levels, ; while the Kruskal-Wallis test was used for the categorical variables with three or more levels, and the Nemenyi test for multiple comparisons. To relate the urinary iodine concentration with the numerical variables, the Spearman correlation was used. 16

The Stepwise method was implemented for the selection of variables for multivariate analysis. The Stepwise method is a mix of the Backward and Forward methods. Thus, first, using the Forward method, the variables with *p* -value < 0.25 were selected for multivariate analysis. Then, the Backward method was implemented, by removing the variable with the highest *p* -value, and repeating the procedure until only significant variables remain in the model. For the Backward method, a 5% significance level was adopted. 17 In the multivariate analysis, linear regression was used.

## Results

A total of 30 pregnant women with a singleton pregnancy and gestational age ranging from 4 weeks and 5 days to 19 weeks and 3 days were evaluated. The average age among them was 27.03 years old, with the minimum age being 18 and the maximum 42 years.

Among them, 13 pregnant women (43.33%) were overweight, 8 (26.67%) had an adequate weight, 6 (20%) were obese, and 3 (10%) were underweight. The weight classification of pregnant women was established through the Technical Standard from the Brazilian Food and Nutrition Surveillance System (SISVAN). 18

The group counted with 15 nulliparous women, 11 primiparas, and 4 multiparas.

The median of urinary iodine concentration samples was 216.73 mcg/l, which is proper for the group, according to WHO. 10 The urinary iodine average (mcg/l) was 247.28 with 95% confidence interval (95% CI = 192.26; 302.29) and standard deviation (SD: 153.35); the minimum value was 75.06, and the maximum was 714.05. In the context of the entire population of pregnant women attended in the prenatal ambulatory of normal risk from the Federal University of Minas Gerais from May to December 2017 (82 patients), for a sample of 30 pregnant women, an error margin of 43 mcg/l would be expected.

The neonatal TSH average (µIU/ml) was 1.4, with the minimum value being 0.29 and the maximum 2.84. The collected data are described in Table 1 .

**Table 1:** Descriptive analysis of numeric variables

Variable	n	Average	Minimum	1 ^st^ Q	2 ^nd^ Q	3 ^rd^ Q	Maximum
Urinary iodine (mcg/L)	30	247.28	75.06	127.65	216.73	309.79	714.02
Neonatal TSH	29	1.4	0.29	0.96	1.22	1.83	2.84
BMI (kg/m ^2^ )	30	26.75	18.4	23.01	26.82	30.22	36.71
Gestational age (days)	30	86.63	33.0	67.0	86.5	108.0	136.0
Age	30	27.03	18.0	22.0	24.5	32.0	42.0

**Abbreviations:** BMI, body mass index; TSH, thyroid-stimulating hormone.

Table 2 shows the relationship between the urinary iodine concentration of pregnant women and the categorical variables. Thus, it is observed that there was a significant difference ( *p* = 0.021) between the urinary iodine concentration of pregnant women and family history of thyroid disease. The average urinary iodine concentration of pregnant women with a family history of thyroid disease was higher when compared with those without ( Table 3 ).

**Table 2:** Relation between the urinary iodine concentration of pregnant women and the categorical variables

Variable	n	Average	S.E.	1 ^st^ Q	2 ^nd^ Q	3 ^rd^ Q	*p* -value
Smoking	29 no 1 yes	250.3 159.8	28.81 -	127.7 159.8	230.3 159.8	309.8 159.8	0.729
Family history for thyroid disease	23 without 7 with	202.2 395.3	19.94 81.76	126.1 280.9	164.7 292.8	278.0 524.8	0.021
Ex-smoker	23 no 6 yes	236.6 302.9	29.83 82.2	127.2 230.3	166.6 268.1	312.7 296.6	0.572
Ex-drinker	13 no 17 yes	221.2 267.2	28.94 44.38	126.8 148.6	164.7 230.3	315.7 292.8	0.786
Parity	15 nulliparous 15 not nulliparous	253.2 241.4	40.57 39.95	137.0 143.7	230.3 203.1	332.9 292.3	0.868
BMI classification	8 adequate 3 underweight 19 overweight	151.8 207.5 293.8	16.66 54.17 39.37	124.3 153.5 156.6	140.2 159.8 291.7	184.9 237.7 352.8	0.071

**Abbreviations:** BMI, body mass index.

**Notes:**^1^ Mann Whitney test; ^2^ Kruskal-Wallis test.

**Table 3:** Relation between the urinary iodine concentration of pregnant women and the numerical variables

Variable/ioduria (mcg/L)	r ^1^	*p* -value ^2^
Neonatal TSH (µIU/ml)	-0.37	0.051
BMI (Kg/m ^2^ )	0.08	0.676
Gestational age	0.2	0.287
Age	-0.11	0.547

**Abbreviations:** BMI, body mass index; TSH, thyroid-stimulating hormone.

**Notes:**^1^ Correlation coefficient; ^2^ Spearman correlation.

The existence of a marginally significant ( *p* = 0.051) and negative (r = -0.37) relationship between the urinary iodine concentration of pregnant women and neonatal TSH was observed ( Table 3 ). In the multivariate analysis, it may be concluded that there was a significant influence ( *p* = 0.023) of the BMI classification over the urinary iodine concentration, and when compared with pregnant women with adequate BMI, those with overweight or obesity BMI showed an increase of 82.74 units in the urinary iodine concentration ( Table 4 ).

**Table 4:** Factors that influence the urinary iodine concentration of pregnant women

Variables	Initial Model			Final Model		
	β	S.E. (β)	*p* -value ^1^	β	S.E. (β)	*p* -value ^1^
Family history for thyroid disease = No	0.00	−	−	0.00	−	−
Family history for thyroid disease = Yes	215.58	81.02	0.014	160.73	81.659	0.060
BMI classification = proper	0.00	−	−	0.00	−	−
BMI classification = underweight	37.82	52.87	0.481	55.75	46.897	0.245
BMI classification = overweight	82.74	28.89	0.009	82.74	34.365	0.023
Neonatal TSH	-43.48	35.11	0.228	−	−	−
VIF	1.223			1.214		
R ^2^	39.04%			26.56%		

**Abbreviations:** BMI, body mass index; TSH, thyroid-stimulating hormone.

**Notes:**^1^ Solid linear regression.

## Discussion

In several countries that have already adopted salt iodination programs, despite normal levels of urinary concentration in schoolchildren, an iodine insufficiency in pregnant women has been shown. 19 20 Studies with American pregnant women also found iodine insufficiency in this subpopulation. 14 In London, Manchester, and Leeds, the median urinary iodine concentration in pregnant women was also below 150 mcg/l, showing the insufficiency of iodine intake for this particular population, even though the United Kingdom's population is generally considered iodine sufficient. 21 This way, the last guidelines from the American Thyroid Association, Endocrine Society, and European Thyroid Association, recommend the administration of iodine supplements to pregnant women and those planning to get pregnant. 3 4 5

Brazilian data on iodine sufficiency in pregnant women are controversial. In 2001, Barca et al. 15 showed iodine sufficiency in 20 pregnant women attended in a public hospital of São Paulo, with iodine excretion represented by the median of urinary concentration of 167.8 mcg/l. In 2008, Soares et al. 22 evaluated 147 pregnant women in Porto Alegre and obtained urinary iodine levels between 22 and 534 mcg/l, with 19.6% of the pregnant women showing urinary iodine concentration lower than 150 mcg/l, and median of 224 mcg/l, that is, adequate levels of urinary iodine concentration. 22

In 2014, in the state of São Paulo, a high rate of iodine insufficiency was observed in pregnant women (median: 137.7 mcg/l). Among the 191 pregnant women, 57% showed urinary iodine concentration lower than recommended by WHO. 23 It is important to emphasize that these three Brazilian studies were carried out when the level of salt iodination was still from 20 to 60 mg iodine/ Kg salt. 24 There are more recent Brazilian studies were carried out in the states of São Paulo and Rio de Janeiro, after ANVISA's resolution of 2013. Saraiva et al. 25 analyzed 629 urine samples from 244 pregnant women in the first 12 weeks of pregnancy in the state of Rio de Janeiro. The median of urinary iodine concentration was adequate (221 mcg/l), with 48.7% of women with insufficiency (<150 mcg/l) and 4.5% with excess (> 500 mcg/l) in at least one of the samples. 25 The median of urinary iodine concentration is used as a populational marker in iodine status. In our study, the concerned population showed iodine sufficiency.

In a study conducted in the city of São Paulo, pregnant women in all the trimesters of pregnancy were evaluated, and a median urinary iodine concentration of 144 mcg/l was found, with rates from 27.3 to 403 mcg/l, which indicates iodine insufficiency. A urinary iodine concentration below 150 mcg/l was found in 52% of pregnant women, and lower than 50 mcg/l in only 2%. The urinary iodine concentration was proper for 44% of the women, and 4% presented with levels above the requirement. 26

Our study corroborates the results found by Saraiva et al. in the state of Rio de Janeiro, 25 which also showed iodine sufficiency in their population. The fact that these two studies have different results from the investigation carried out in São Paulo by Mioto et al. 26 may be justified by the time of the urinary iodine samples collection (in the afternoon in our study, three random samples in the study from Rio de Janeiro, and a morning sample in São Paulo). The urinary iodine release levels reflect the recent iodine intake, and the morning period is when iodine concentration is at its lowest. This way, studies carried out using urine collected in morning periods cannot be directly compared to studies in which urine samples were collected throughout the day. 27

The main limitations of this study are its small sample size and the lack of information on dietary intake. However, until this moment, our research is one of the few Brazilian studies evaluating the iodine nutritional status in pregnant women while correlating it with the thyroid function of newborns, an outcome that is one of the primary and most concerning consequences of iodine deficiency in pregnant women. Therefore, despite the limitation of the small sample of pregnant women, no neonatal TSH was higher than 5 mIU/l in the studied sample, showing iodine sufficiency in this population sample.

In our results, overweight was associated with iodine dosages above the normal. Similarly, the study carried out by Saraiva et al. 25 also found that correlation. This result may be explained due to higher food intake with higher levels of iodine by this subpopulation.

This study shows that the population of pregnant women attended in the prenatal ambulatory of normal risk from the Federal University of Minas Gerais is considered iodine sufficient, even after the decrease of salt iodine levels in Brazil. It should be emphasized that there were no cases of neonatal hypothyroidism reported in the study. In this context, we may state that there is insufficient data to recommend the iodine supplementation for all Brazilian pregnant women, in the face of the heterogeneity of studies' results. Brazil is a continental country, which makes uniform standardization hard to be implanted. Several factors may interfere in the iodine sufficiency of pregnant women. Differences in the diet, social and demographic characteristics, easiness of people and food transportation, business negotiations, and processed food consumption may explain the coexistence of areas with iodine insufficiency and excessive iodine intake in the country. 28

## Conclusion

The results of this study reinforce the importance of regional studies, such as this one. Studies in different regions and the collection of a higher number of urinary samples per pregnant woman might improve the accuracy of the results, considering that urinary iodine concentration may vary day-to-day, including within-day variation. 29 30 This way, it is too early to recommend a universal iodine supplementation for Brazilian pregnant women, and more studies need to be carried out. It is worthy to emphasize that iodine supplementation for pregnant women in iodine sufficient areas is associated with the risks of the fetus's excessive exposure to iodine.
